# Emergency room presentations of people with anorexia nervosa

**DOI:** 10.1186/s40337-023-00742-x

**Published:** 2023-02-09

**Authors:** Philip S. Mehler, Kristin Anderson, Maryrose Bauschka, Jeana Cost, Asma Farooq

**Affiliations:** 1 ACUTE Center for Eating Disorders at Denver Health, Denver, CO USA; 2 University of Colorado School of Medicine, Denver, CO USA; 3 University of Utah School of Medicine, Salt Lake City, UT USA; 4 Eating Recovery Center, Denver, CO USA

**Keywords:** Emergency room, Medical complications, Electrolyte, Pain, Hypoglycemia

## Abstract

People with anorexia nervosa (AN) tend to shy away from engaging in typical primary care provider relationships in order to avoid detection. Therefore, they may seek care for their medical concerns through a local emergency department (ED). Inherently, AN is associated with a litany of medical complications, which become more prevalent as the severity of their eating disorder increases. Notwithstanding the typical young age at the onset of AN, no body system is immune to these medical complications. Thus, ED providers may need to pursue a medical diagnosis in order to explain presenting symptoms in people with AN. In addition to the medical issues, AN is also a serious mental illness with high mortality rates, including deaths by suicide. Therefore, ED providers also need to be familiar with relevant mental health issues for these people.

## Plain English summary

People with anorexia nervosa frequent emergency departments to obtain their medical care. It is thus important for emergency department personnel to be familiar with this increasingly common and serious disorder. As opposed to most other mental illnesses, anorexia nervosa is associated with many dangerous medical complications, which become more problematic as the malnutrition and weight loss become more severe. All body systems are adversely affected. The mortality rate of anorexia nervosa is the second highest of all mental disorders, with medical complications and suicide being the top two causes of death. Mandated medical care may occasionally be required to obtain ongoing treatment for people with anorexia nervosa when they present to the emergency department with severe malnutrition and other emergent complications.

## Introduction

People with anorexia nervosa (AN) tend to try and hide their emaciated state. Thus, they may shy away from having a well-established primary care relationship and instead they may use emergency departments (ED) in lieu thereof when medical concerns arise [1]. A study, from an academic medical center’s university hospital, demonstrated that nearly one out of six adolescent patients in their ED had a covert eating disorder related-medical issue as the cause for their visit [2]. Moreover, because people with AN, especially as the AN becomes increasingly severe, often have medical complications which can affect every body system [3], it is understandable why they may therefore present for medical care in EDs [4]. Therefore, it is valuable to be familiar with the urgent medical conditions which can necessitate an ED encounter for people with both subtypes of AN, namely AN-restricting (AN-R) and AN-binge purge (AN-BP) [4–6]. A very recent study demonstrated that individuals with AN often had a recent preceding outpatient electrolyte abnormality, and thus the ED may uncover covert eating disorders and identify the need for eating disorder follow-up [7]. In addition, these people can end up in the ED with life-threating malnutrition and involuntary care may be required as a life-saving intervention. It is therefore also worthwhile to understand how to navigate these types of serious mental health situations which may manifest during their stay in the ED. In the past decade there have been reviews of the presentation of people with AN in an ED setting [8]. This paper updates and adds new information to this literature. Specifically, new information is presented on COVID-19, cardiac complications, sarcopenia, and hypoglycemia, and there is expanded and new information regarding mental health aspects of ED presentations.

### Emergency psychiatric issues

By utilizing the ED for medical care, it typically gives people with AN the flexibility to see busy and different providers who only focus on the most imminent issue at hand. For some patients, their lack of a PCP is due to the providers themselves not being comfortable seeing a patient with active AN, whether that is due to their lack of expertise with the medical complications of AN or due to the severity of the patient’s comorbid psychiatric illness.

Psychiatric comorbidities among people with AN are very prevalent and can commonly land someone in the ED [9]. More recently, since the start of the COVID-19 pandemic, there has been an uptick in people with AN utilizing EDs due to mental health emergencies. The number of such visits, among adolescent females specifically, doubled during the pandemic [10]. In addition, there has been a significant COVID-19-related increase in outpatient volume of people with eating disorders seeking care [12]. The most common psychiatric comorbidities are depressive disorders and anxiety disorders (69%)—specifically obsessive–compulsive disorder and social anxiety [11]. The presentation of these psychiatric issues can take the focus off the underlying AN and associated medical complications.

In addition, people with AN are brought to the ED in a variety of ways, including emergency vehicles, family escort and provider escort such as by an “interventionist.” In the United States, the use of interventionists is growing in the field of eating disorders due to the high level of resistance that people with AN tend to manifest. Interventionists are mental health professionals who support and provide direction to families whose loved ones are struggling with severe eating disorders. If the person with AN has not been medically evaluated recently or appears to have immediate health concerns, the interventionist will in turn likely utilize an ED for an assessment prior to guiding the individual to an eating disorder treatment facility.

Another new mental health issue for this population involves the term “terminal AN.” While criteria for “terminal AN” have recently been proposed and may be cited in the ED as justification for refusal of life-saving treatment, it is crucial to recognize that at this time, there are no evidence-based criteria to support a diagnosis of “terminal AN” [13–15]. Data suggest that recovery can occur even after decades of this illness [16]. Malnutrition itself is not a terminal illness, since all complications of AN-induced malnutrition are reversible with nutrition and appropriate supportive medical care, with the well-established exception of bone mineral density loss and the possible exception of reductions in brain cortical surface volume [17]. ED providers need to be aware of this new complex ethical issue as it may arise when people with extreme forms of AN access care in the ED.

Malnutrition has been shown to affect the brain in a variety of ways including causing increased obsession with food, depression, apathy, and impulsivity, all of which can reinforce AN cognitions and cloud decision-making [18–20]. And, ambivalence regarding treatment is common, which may adversely impact an individual’s decision-making regarding necessary treatment for their AN [21–24].

The achievement of a healthy weight is the strongest predictor of recovery in AN, which often requires intensive in-patient treatment [25, 26]. While it is likely ideal for patients to voluntarily seek care for their AN, if safety and capacity are in question, involuntary treatment can be lifesaving. Involuntary treatment can be distressing for both providers and patients, but if effective, it may ultimately be regarded by patients as having been a necessary and life-saving intervention [27–29]. Therefore, it would be important to ensure that, if a people with severe AN were to refuse life-saving treatment in the emergency room, that a safety assessment and capacity evaluation are completed timely.

It has long been believed that mental capacity of patients with AN is impaired, especially for those experiencing severe degrees of malnutrition. Capacity in a given moment is not only influenced by cognitive and rational functioning, but also by a multitude of emotional factors as well. People with AN tend to have a higher level of subtle impairments in mental capacity than that noted in other psychiatric disorders, which can make the capacity assessment even more complex. Often, individuals with AN are quite articulate, and are able to identify the risks and benefits of a proposed treatment, but will struggle to appreciate the consequences of their choices [33].

In general, people with mental illness who lack capacity are often unwilling to accept the severity of their illness and resist and refuse treatment, which can lead to life-threatening outcomes. It is therefore helpful for an emergency medicine provider to be aware of the laws, policies and procedures available in their locale when a person with AN is identified to lack capacity. In the United States, all 50 states have statutes regarding civil commitment for psychiatric disorders. These statutes vary significantly from state to state and from country to country. In the United States, eight states allow civil commitment only for the criteria of dangerousness i.e. person must demonstrate an immediate, physical danger to self or others before a court can intervene and order treatment. The remaining 42 states include additional criteria for grave disability, which usually means a condition in which the individual, because of a mental disorder, is in danger of serious physical harm due to a failure to provide for their own essential human needs, such as food, clothing or shelter [34].

Individuals with a prolonged history of AN, are much more likely to have previously attempted suicide and face a significantly elevated likelihood of considering it in the future. Unfortunately, suicide is a frequent cause of death in patients with both AN and bulimia nervosa (BN), often as high as 20% [30]. Therefore, it is also important for emergency medicine providers to screen for suicidality in patients with AN [31, 32]. A cutoff score of ≥ 4, on the eight-item Patient Health Questionnaire (PHQ-8), has previously been shown to be effective in screening for major depression (73% sensitivity, 94% specificity) [5].

Finally, once a person with AN has been identified as being in need of follow-up ED care, there are several options for care that a provider needs to consider. Identifying the correct level of care can be a challenging task as there are many variables that need to be considered such as severity of illness, insurance coverage, program availability and patient age and gender. Overall, there are 6 levels of care which are recognized within the eating disorder industry, and they vary by the level of support that they can provide [34, 35]. The levels of care available, in order from least intensive to most intensive, are: (1) Outpatient, (2) Intensive Outpatient, (3) Partial Hospitalization, (4) Residential, (5) Behavioral Health Inpatient and (6) Medical Inpatient. It is prudent that a clinician with AN knowledge provide an eating disorder assessment to determine the optimal next phase of care.

## Medical complications

### Cardiac

All body systems are at risk for developing serious complications as the AN becomes increasingly severe (Tables 1, 2). Cardiac complications are prominent amongst them. There are several cardiovascular changes the clinician should be aware of when evaluating people with AN, starting with vital signs. Patients typically present with bradycardia (heart rate < 60) and hypotension (systolic blood pressure < 90 mmHg). Sinus bradycardia may be the most common presenting sign of anorexia nervosa, present in up to 95% of patients [36], and is thought to be due to increased vagal tone in attempt to conserve energy [37]. Hypotension results from decreased cardiac contractility caused by decreased cardiac muscle mass [38]. Sinus bradycardia is usually asymptomatic; however, it is reasonable to recommend admission to a telemetry unit for patients with a resting heart rate < 40 beats per minute, bradycardia not of sinus origin, or an episode of recent syncope. Clinicians should also note that in the rare cases of symptomatic bradycardia, temporary pacing should only be considered in the setting of life-threatening hemodynamic instability [39], since the bradycardia will resolve with early refeeding and rewarming. Because bradycardia is so common in AN, finding a heart rate much above 60 should at least alert the clinician to the small possibility of an underlying medical complication, such as occult infection or a cardiopulmonary process.

**Table 1:** Medical complications of anorexia nervosa

Cardiovascular	Endocrine and metabolic
Bradycardia and hypotension	Amenorrhea
Mitral valve prolapse	Unintended pregnancy and miscarriages
Sudden death—arrhythmia	Osteoporosis
Refeeding syndrome	Thyroid Abnormalities
Echo changes	Hypercortisolemia
Pericardial effusions	Hypoglycemia
	Diabetes insipidus
**Dermatologic**	Hypophosphatemia
Dry skin	
Alopecia	**Hematologic**
Lanugo hair	Pancytopenia
Starvation-associated pruritis	Decreased sedimentation rate
Acrocyanosis	
	**Neurologic**
**Gastrointestinal**	Cerebral atrophy
Constipation	Thalamic dysfunction
Refeeding pancreatitis	
Acute gastric dilatation	**Pulmonary**
Delayed gastric emptying	Aspiration pneumonia
Hepatitis	Respiratory failure
Dysphagia	Spontaneous pneumothorax
Superior Mesenteric	Emphysema
Artery (SMA) syndrome	
	**Ear-Eyes**
	Lagophthalmos
	Patulous Eustachian Tube Dysfunction

**Table 2:** Medical complications of anorexia nervosa—binge purge

Gastrointestinal	Cardiac
Dental erosion and caries	Arrhythmias
Parotid gland swelling	Diet pill toxicity
Esophageal rupture	Palpitations
Gastroesophageal reflux (GERD)	Emitene cardiomyopathy
Constipation due to laxative abuse	
Rectal prolapse	**Endocrine**
Mallory-Weiss tear	Irregular menses
	Mineralocorticoid excess (aldosterone)
**Pulmonary-Mediastinal**	Diabulimia
Aspiration pneumonitis	
Pneumomediastinum	**Metabolic**
	Hypokalemia
**Opthalmic**	Dehydration
Scleral hemorrhage	Nephropathy
	Metabolic alkalosis
**Ear Nose Throat** (ENT)	Pseudo Bartter's syndrome
Epistaxis	
Pharyngitis	**Dermatologic**
	Russel’s sign
	Edema

For many years the teaching has been that QTc prolongation is common and inherent to people with AN, and that malignant ventricular arrhythmias are the cause of the often-reported sudden cardiac death. This is progressively changing. Recent research has more definitively shown that QTc prolongation is not an intrinsic feature of AN [40], and many only be present in 1–3% of people with AN [40]. Therefore, if QTc prolongation is identified, the clinician should first look for extrinsic causes including electrolyte abnormalities (most commonly hypokalemia) and medication side effect. Patients with a QTc > 500 ms should be admitted for telemetry monitoring as their risk for dangerous torsades de pointes (TdP) goes up markedly [41]. The LACE-AN pilot study, which looked at more than 10 patient years of monitoring via an insertable cardiac monitor in people with severe AN, surprisingly did not identify ventricular tachyarrhythmia in any patient [43]. Rather, bradycardia and sinus pauses were identified as the most common rhythm abnormalities. A new conceptual model, that includes four key patho-biological changes that may be responsible for the increased mortality risk in people with AN, has therefore recently been proposed and includes the following: (1) cardiac structural changes leading to acute heart failure (2) purging behaviors causing hypokalemia and other electrolyte abnormalities, along with increased aldosterone levels due to dehydration from chronic purging, with resultant TdP (3) cardiac autonomic dysfunction, which predisposes to sudden death and (4) bradyarrhythmias which may lead to pulseless electrical activity (PEA) [44].

Patients with AN commonly have mitral valve prolapse as chronic starvation causes left ventricular (LV) atrophy, decreased LV mass, and decreased chamber volumes, the latter of which leads to annular laxity with resultant mitral valve prolapse. Mitral valve prolapse, which may be present in up to one-third of people with AN, is typically associated with palpitations and resolves with weight restoration. Pericardial effusion is another structural abnormality recently identified with AN, occurring in up to 25% of patients. Like mitral valve prolapse, pericardial effusions are typically asymptomatic, remit with weight restoration, and can be followed as an outpatient, unless the rare tamponade is diagnosed [41]. Takotsubo cardiomyopathy can also be seen in patients with AN, and may be attributable to the stress of the psychological condition plus severe malnutrition. However, the cardiac ejection fraction is generally normal in people with AN; only 15% had a reduced ejection fraction in one reported series [42].

In people with AN-BP, purging behaviors result in hypokalemia in about 40% of patients and are a common cause of cardiac problems [40]. These people rarely may abuse syrup of ipecac to stimulate vomiting. Ipecac contains the alkaloid emetine, which is toxic to cardiac muscle, and may cause dysrhythmias and heart failure [45]. In people with AN, who present in a sympathomimetic state, their clinicians should have a suspicion for diet pill toxicity. Culprits may include Ma Huang (Ephedra), caffeine, or FenPhen^R^, all of which can cause serious cardiac dysrhythmias. Chronic FenPhen ^R^ use may lead to valvular disease and primary pulmonary hypertension [46].

### Pulmonary complications

Aspiration pneumonia, pneumomediastinum, and spontaneous pneumothorax should all be considered in people with AN presenting with respiratory complaints or chest pain. These are all fairly rare, occurring in less than 1% of cases [47]. Aspiration pneumonia can result from self-induced vomiting in people with AN-BP or may be a result of dysphagia due to starvation induced weakening of the pharyngeal muscles. Spontaneous air leak syndromes are thought to be associated with decreased lung surfactant production and emphysematous-like changes that are seen in prolonged starvation. These changes cause the lung tissues to be more susceptible to injury. Patients who purge via vomiting are also predisposed to developing pneumomediastinum and pneumothorax due to the increased intrathoracic pressure associated with vomiting [48]. If symptomatic, patients with pneumomediastinum most commonly present with chest pain, followed by persistent cough and sore throat [49]. Patients typically do not require specific treatment, though they should be admitted for observation. Spontaneous pneumothorax may also occur in people with AN [50]. However, pneumothorax may be difficult to resolve in people with AN as the lung may remain collapsed for an extended period of time until their state of malnutrition resolves [51].

### Gastrointestinal

People with both AN-R and AN-BP can develop abdominal pain which necessitates an ED encounter. One cause of abdominal pain in people, with both AN-R and AN-BP, is superior mesenteric artery (SMA) syndrome. While its frequency is unknown, it is known to develop as a direct result of weight loss and resultant atrophy of the mesenteric fat pad which normally tethers the SMA in place. Absent the fat pad, the SMA moves medially and compresses the third portion of the duodenum causing a small bowel obstruction. SMA-syndrome generally develops in those with body mass indices (BMI) less than 16 mm/kg^2^ and presents with severe epigastric pain fifteen minutes after eating, which is resolved by vomiting [51]. The diagnosis is best made by obtaining an abdominal CT scan or an upper GI series, and specifically alerting the radiologist that the test is being obtained to rule-out SMA syndrome. Treatment is based on weight restoration and reconstitution of the fat pad via a soft liquid diet [52]. Surgery is rarely ever indicated [53]. Another cause of abdominal pain, mostly found, again, in those with more severe forms of AN, is acute gastric dilatation. This idiopathic and overall rare condition is characterized by the sudden onset of marked gastric dilatation with affected people presenting with left upper quadrant abdominal pain, obvious distention and a tympanitic abdominal examination. Diagnosis is confirmed by obtaining an abdominal radiograph which demonstrates marked gastric dilatation [54]. Treatment is based on rapid gastric decompression by insertion of a nasogastric tube, making the patient nothing per oral (NPO), correction of aberrant serum electrolyte levels and admission to the hospital. Some believe that acute gastric dilation is a sequelae of SMA syndrome [55].

Constipation is another reason why both subtypes of people with AN might seek ED care. All people with AN have markedly prolonged colonic transit time as a result of their weight loss and are at risk for constipation [56], occurring in approximately 30% of people with AN [57]. Thus, these patients can suffer with constipation especially those with more severe AN at the beginning of refeeding. But, more commonly, this is a problem for the AN-BP patients who not only have intrinsic slowed colonic transit, but if their purging behaviors involve stimulant laxative abuse, are at risk for the sequelae termed, “cathartic colon syndrome” [58]. This entity is likely attributable to a direct toxic effect of the stimulant laxatives (e.g. senna, bisacodyl, phenolphthalein) on the myenteric and Auerbach’s plexus in the colonic wall. After prolonged and excessive exposure to said stimulant laxatives and damage to the nerve plexus, the colon can be converted into an inert tube, devoid of peristalsis and incapable of propagating fecal material resulting in obstruction [59]. Obtaining an abdominal radiograph would be diagnostic.

People with more severe forms of AN may present to the ED with a complaint of dysphagia. This results from marked atrophy and weakening of the pharyngeal muscles, resulting in dysphagia and placing them at risk for aspiration pneumonia [59]. A modified barium swallow would reveal this etiology. It can be treated by a speech therapist and a temporary modified soft diet. Also, related to the gastrointestinal system, there are a number of case reports of pancreatitis, both as a result of starvation as well as in the early phases of refeeding [60]. The presentation is of typical pancreatitis symptoms [61].

Finally, about a third of these people may be noted to have elevated liver transaminases on a complete metabolic panel blood test [62] As the BMI falls, this is increasingly common. Elevations of the serum ALT and AST, in the range of levels in the hundreds, is often noted and is likely as a result of programmed death of hepatocytes, referred to as apoptosis or autophagy [63], due to starvation. While there are certainly other potential causes of these enzyme elevations, if due to AN, the ALT is generally more elevated than the AST, and bilirubin, and alkaline phosphatase are generally not affected by the starvation of AN [64]. The transaminase elevations, by themselves, are not a reason for admission, if close outpatient follow-up and serial lab draws are available while refeeding is continuing, as they typically will revert to normal with informed refeeding and weight gain.

Profound diarrhea might cause a person with AN to seek an ED evaluation. Certainly, common is common, and therefore an evaluation for infectious causes is indicated. However, AN itself can result in diarrhea, early on in the refeeding process, due to loss of absorptive area in the small bowel and a dumping-type syndrome can develop as a result. Diagnostically, a serum diamine oxidase level can be obtained and will be found to be low in AN when the diarrhea is due to early refeeding and malabsorption [65].

### Endocrine

The endocrine system is greatly disrupted by AN [66]. Aside from amenorrhea which is commonly found (88%), other complication such as diabetes insipidus, are relatively rare [67]. The most serious endocrine complication, found in about 10% of both types of AN [40], is hypoglycemia [67]. As the BMI falls, and certainly as it nears 10 kg/m^2^ or less, this becomes more prevalent. It is a direct result of starvation and weight loss with resultant depleted hepatic energy stores and an inability to activate gluconeogenesis and glycogenolysis [68]. Moreover, as opposed to diabetics who can generally perceive the onset of hypoglycemia after they start to develop the typical symptoms, such as hunger, agitation, diaphoresis and nausea, people with AN lack these neuroglycopenic symptoms [69]. Therefore, they are at risk for progressive hypoglycemia, seizures, coma and death. There is an evolving theory that covert hypoglycemia may be a more common cause of sudden death in AN than heretofore recognized [44]. When a person with AN presents to the ED due to hypoglycemia, they should be admitted because the usual treatments to raise the blood sugar can result in a paradoxical exuberant release of insulin and cause the hypoglycemia to quickly recrudesce. Slow nasogastric feeds may be the best treatment option early on in this scenario, with frequent point of care glucose testing.

### Renal and electrolytes

Because people with AN may use the ED as their defacto primary care site, it is not uncommon for electrolytes to be drawn as part of an evaluation. In the patients with AN-R, their serum electrolytes should generally be normal, and if hypokalemia or a metabolic alkalosis is present, it is a sine qua non that they are more likely of the AN-BP subtype. The electrolyte exception to this rule is that patients with AN-R may present with severe hyponatremia in 15% of cases [70]. The reason for this is that as a result of their starved state, their kidneys are unable to clear free water. Therefore, they can develop critical hyponatremia with rapid ingestion of just a few liters of water versus a normal-weighted person who can safely drink many more liters of water [71]. People with AN are known to water-log in an attempt to falsely elevate their weights around the time of their appointments with their treatment team. Therefore, they may present to the ED seeking care for symptoms and signs of hyponatremia including, nausea-vomiting, altered mental status and seizures. This hyponatremia is not due to the syndrome of inappropriate antidiuretic hormone secretion (SIADH), although SIADH is also described in AN [72].

In reference to patients with AN-BP, they may also present to the ED seeking treatment for abnormal electrolyte symptoms which develop as a direct result of their purging behaviors. As with patients with AN-R, hyponatremia can be from “excessive” free water intake or, more likely, especially with AN-BP it is hypovolemic hyponatremia, as a result of dehydration from laxative or diuretic abuse or from self-induced vomiting [31]. These two major causes of hyponatremia can be differentiated by a combination of physical findings, urine electrolytes and osmolality and the clinical setting. The etiology of hyponatremia is important to define because the treatments vary greatly from slowly replacing volume as normal saline versus restricting free water intake. But, with all cases of hyponatremia in people with AN, correction is to be done with extreme caution, given the small volume of distribution in people this size, and in order to avoid the devastating consequences of rapid sodium correction, known as central pontine myelinolysis (CPM), which has also been reported in people with AN [70].

The most frequent chemical aberrations which are associated with AN-BP are hypokalemia (42%), and metabolic alkalosis (33%) which can be critically abnormal [40]. Hypokalemia is seen as a result of all three modes of purging and may present with muscle weakness, palpitations or an ileus. With self-induced vomiting most of the potassium loss is in the urine since gastric secretion contains only 5–10 mEq of potassium per liter. The vomiting results in a metabolic alkalosis that produces a bicarbonate diuresis, obligating potassium as the accompanying cation loss [73]. Gastrointestinal potassium loss, as a result of laxative abuse, largely directly results from colonic potassium loss in the diarrhea. Also, although typically, a decrease in serum potassium from a serum level of 4 mEq/L down to 2 mEq/L reflects approximately a 200–400 mEq loss of potassium, it is important to note with diuretic abuse or self-induced vomiting, and their attendant metabolic alkalosis, that the total body potassium deficit is not as great because some of the hypokalemia is not true potassium loss, but rather an alkalosis-based redistribution of hydrogen ions moving out of cells and potassium moving into cells. As the alkalosis is treated, potassium goes back into the serum and helps to raise the potassium level back to normal [74]. Thus, the amount of supplemental potassium required is less than one might predict. In contrast, with laxative abuse, and its attendant hyperchloremic metabolic acidosis, any degree of hypokalemia is even more profound, because the acute acidosis should have driven potassium out of cells in exchange for hydrogen ions, and as this is corrected, potassium will go back into cells into the serum and necessitate more supplemental potassium than one might predict.

Finally, the aforementioned metabolic alkalosis, seen with both self-induced vomiting and diuretic abuse, is commonly found on the electrolyte panels of patient with AN-BP who come to the ED because of weakness, palpitations or syncope. It is attributable to volume contraction and dehydration from purging [75]. A spot urine chloride determination, prior to intravenous saline therapy, is the most helpful laboratory test in determining the cause of the metabolic alkalosis. If the urine chloride concentration is less than 10 mEq/L, it is pathognomonic for dehydration-induced contraction alkalosis [76]. However, it is important that ED providers are restrained with the rapidity in which they administer intravenous saline to correct the severe degrees of metabolic alkalosis in people with AN-BP. Because of the chronic state of dehydration found in AN-BP, there is ongoing stimulation of adrenal aldosterone secretion, to prevent hypotension a fainting, resulting in a salt-avid state. This high serum level of aldosterone can result in rapid and marked edema formation if the intravenous saline is delivered at a fast rate. Rather, these patients with marked metabolic alkalosis (serum bicarbonate > 34 mmol/L) are best initially admitted to an observation unit to be given intravenous saline at a slower rate (50–100 mL/h) to avoid causing marked Pseudobartter’s edema [77] and troubling accompanying weight gain of 10–20 pounds over just a few hours of time [78].

### Musculoskeletal

Although AN typically affects people in their late teens and twenties [79], nevertheless these people suffer from a number of musculoskeletal complications which are generally seen in an older population. Specifically, as a result of low levels of leptin, sex hormones and insulin growth factor-1 (IGF-1), along with growth hormone resistance and elevated cortisol levels, a large percent (30–90%) of people with AN, are at high risk for severe loss of bone mineral density and the development of osteoporosis with resultant fragility fractures [40, 80, 81]. In fact, loss of bone mineral density, which begins very soon after the onset of AN, even in people in their teenage years, may cause permanent harm and is not easily reversible even with nutritional rehabilitation and weight restoration [82]. Therefore, when a patient with AN presents to the ED with back or joint pain, even in the absence of major trauma, there should be a low threshold to obtain imaging radiographs as part of their evaluation. Fragility long bone fractures, vertebral compression fractures, and rib fractures may be caused by minor trauma and could be missed given the relatively young age of this patient population. Also because of the sarcopenia of AN, they are at risk for mechanical falls [83].

Finally, other miscellaneous complications, some major and others more minor, can be present on examination and noted when they present to the ED. These include, in AN-BP, conjunctival hemorrhages, epistaxis, esophageal perforation, gastroesophageal reflux disease (GERD), patulous eustachian tube dysfunction and hearing loss, corneal abrasions from lagophthalmos, marked leukopenia and neutropenia, thrombocytopenia and anemia [81] (Table 1). In the AN-R subtype, many of these same complications can be present aside from the ones directly related to purging behaviors.

## Conclusion

In summary, due to the inherent causal relationship between AN and the aforementioned medical complications, people with AN frequent the ED seeking care for these problems. Although there certainly can be a functional component to some complaints, ED caregivers should consider pursuing an organic cause to explain presenting symptoms given the reality that serious medical complications become increasingly prevalent as the severity of the AN increases. Further, because of the high lethality of AN, issues regarding involuntary ongoing treatment often first arise during an ED stay.

## Data Availability

Not applicable.
